# Extensive HIV-1 Intra-Host Recombination Is Common in Tissues with Abnormal Histopathology

**DOI:** 10.1371/journal.pone.0005065

**Published:** 2009-03-31

**Authors:** Susanna L. Lamers, Marco Salemi, Derek C. Galligan, Tulio de Oliveira, Gary B. Fogel, Sara C. Granier, Li Zhao, Joseph N. Brown, Alanna Morris, Eliezer Masliah, Michael S. McGrath

**Affiliations:** 1 BioInfoExperts, Thibodaux, Louisiana, United States of America; 2 Department of Pathology, Immunology, and Laboratory Medicine, University of Florida, Gainesville, Florida, United States of America; 3 Department of Laboratory Medicine, Positive Health Program, University of California San Francisco, San Francisco, California, United States of America; 4 South African National Bioinformatics Institute, University of the Western Cape, Cape Town, South Africa; 5 Natural Selection, Inc., San Diego, California, United States of America; 6 The Department of Toxicology, Shandong University, Jinan, China; 7 Pathologica Inc., Burlingame, California, United States of America; 8 Department of Pathology, School of Medicine, University of California San Diego, San Diego, California, United States of America; 9 AIDS and Cancer Specimen Resource, San Francisco, California, United States of America; University of Nebraska, United States of America

## Abstract

There is evidence that immune-activated macrophages infected with the Human Immunodeficiency Virus (HIV) are associated with tissue damage and serve as a long-lived viral reservoir during therapy. In this study, we analyzed 780 HIV genetic sequences generated from 53 tissues displaying normal and abnormal histopathology. We found up to 50% of the sequences from abnormal lymphoid and macrophage rich non-lymphoid tissues were intra-host viral recombinants. The presence of extensive recombination, especially in non-lymphoid tissues, implies that HIV-1 infected macrophages may significantly contribute to the generation of elusive viral genotypes *in vivo*. Because recombination has been implicated in immune evasion, the acquisition of drug-resistance mutations, and alterations of viral co-receptor usage, any attempt towards the successful eradication of HIV-1 requires therapeutic approaches targeting tissue macrophages.

## Introduction

The intra-host evolution of HIV-1 is characterized by the ability of the virus to generate, on a daily basis, an extensive sequence diversity due to the high mutation rate of reverse transcriptase (3×10^−5^ substitutions per site per generation) [1], rapid viral turnover (10^−8^ to 10^−9^ virions per day) [2]–[4], large number of infected cells (10^7^ to 10^8^ cells) [5], and recombination [6], [7]. Recombination plays a significant role in generating genetic variation in the HIV-1 genome [8] and it has been shown that the mean recombination rate can be up to 5.5 times greater than the mean mutation rate [9]. Recombination can occur when cells are super-infected with different viral strains [10], leading to exchanged genetic segments in the progeny virus and the rapid generation of completely novel and elusive genotypes within the infected individual [9], [11]–[13]. Laser micro-dissection studies have shown that individual cells are able to harbor up to ten unique viruses [12]. Recombination is, therefore, one of the most dramatic means for a virus like HIV to generate diversity and it has been implicated in immune evasion and escape [11], [14], the potential to generate drug-resistance mutations [15], [16], and the switch of co-receptor usage from CCR5 to CXCR4 [17], [18]. However, the extent of viral recombination in different lymphoid and non-lymphoid tissues infected *in vivo* by HIV-1, as well as the relationship between recombination and pathogenesis in AIDS patients, are currently unknown.

The causes of death for HIV-infected individuals are numerous and have changed since the introduction of highly-active antiretroviral therapy (HAART), although several AIDS-defining illnesses, including non-Hodgkin's lymphoma, dementia, *Pneumocystis carinii* infection, atypical mycobacteria infection, and brain toxoplasmosis, still persist. In the United States, the incidences of non-AIDS defining diseases, such as HCV infection, non-AIDS-defining lymphomas, cardiovascular disease, liver dysfunction, and splenomegaly have also increased along with the life span of HIV-infected individuals [19]. Although T-cell depletion is characteristic of AIDS, macrophage infection is also an important component during the progression of HIV infection to AIDS. In the case of AIDS dementia, macrophages and microglia are the primary immune cells causing destruction of neuronal tissues. In the case of AIDS related lymphomas, it has been hypothesized that macrophages may be B-cell mitogens, thus contributing to the development of the disease [20]. In sheep, the Maedi-Visna virus infection, which targets macrophages and dendritic cells but not T-lymphocytes, also leads to progressive diseases and death that resembles the wasting and brain diseases of HIV without the T-cell immunodeficiency [21]. In HIV-infected humans, tissue-based abnormalities discovered at autopsy could be linked to long-term HIV infection or toxicity associated with the continued use of antiretroviral drugs; therefore, the characterization of HIV-1 variants infecting tissues with abnormal histopathology may shed light on the important relationship between viral diversity and pathogenesis.

In the present study, we examined 780 HIV-1 envelope gp120 sequences amplified from lymphoid and non-lymphoid tissues displaying different degrees of histopathology from seven patients who died with a variety of end-stage HIV-associated diseases. Four of the patients were on HAART therapy near or at the time of death. The comparison of brain and lymphoma tissues to lymphoid tissues allowed the evaluation of macrophage-localized HIV (brain) as compared tissues that may contain a mixed population of macrophage and T-cell associated HIV tissues (lymphoid tissue). By using a number of robust statistical tests to detect intra-host recombination, we identified the presence of recombinant sequences in different tissues and correlated the extent of recombination with precise details relating to autopsy and tissue pathology reports.

## Results

### Case studies and tissue histopathology

HIV-1 envelope gp120 sequences were amplified successfully from 53 normal and abnormal tissues collected *post mortem* from seven patients who died of AIDS. Two patients (CX and GA) had progressive HIV-associated dementia (HAD), three died of non-Hodgkin's lymphoma (AM, IV, BW), and two of systemic infections (AZ, DY). Extensive details about each case study are included in the Supporting Material S1. The autopsies were performed at various locations in the United States from 1995 to 2003. Although every attempt was made to amplify sequences from identical tissues from all patients, this was impossible due to several factors, including the fact that many brain tissues, especially from those patients without extensive brain disease, contained no amplifiable HIV.

Tissues were grouped and analyzed according to 1) histopathology: 11 normal tissues vs. 39 abnormal tissues (three tissues did not have an associated histology report) or 2) in terms of the primary disease of the infected subjects, such as dementia (14 tissues from subjects CX and GA), lymphoma (26 tissues from subjects IV, AM and BW), and other complications (8 tissues from subject DY who died from *Mycobacterium avium complex* infection and 5 tissues from subject AZ who had severe cardiovascular disease, including atherosclerosis in brain tissues and chronic hepatitis C infection).

**Figure 1:**
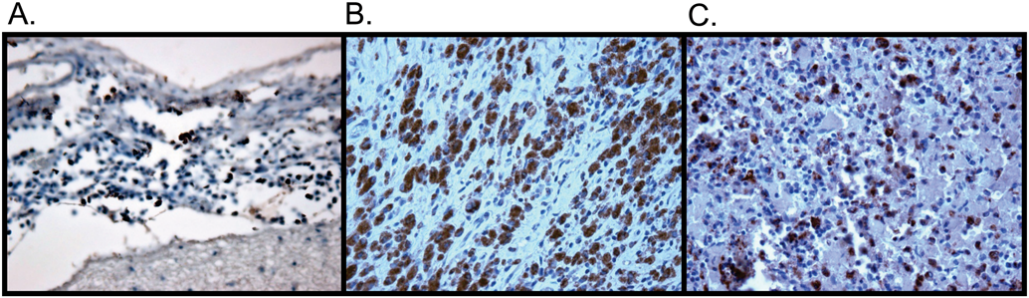
Histopathology. Anti-CD68 staining of A) meninges from patient CX; B) large cell lymphoma from AM; and C) spleen from patient DY. CD68 positive cells are stained brown.

Figure 1 shows three examples of macrophage-specific CD68 staining in meninges, lymph node and spleen from subjects with dementia, lymphoma and systemic infection respectively. In general, most tissues were highly positive for CD68 staining (tissue macrophages), and moderately positive for p24 staining. When present, the p24 stained cells were only tissue macrophages (data not shown). CD68 staining would be negative in normal brain tissues. Finding these cells in the brain at the same frequency as in the spleen and lymph nodes highlights the importance of the frequency and potential importance of CD68 macrophages in the pathogenesis of brain disease. The p24 co-localization observed in the brain was only found in the CD68 cell population. This, therefore, provided strong evidence that at least a subpopulation of the abnormally present macrophages expressed virus and would be in an activation state. No Mac387 staining was present in pathologic brain and lymphoma tissues suggesting that the CD68 expressing cells present in those tissues were relatively long lived.

### Variable frequency of HIV-1 intra-host recombination in different tissues

Recombinants were identified using two methods. The first method, described in detail in Salemi et. al. [22], was specifically designed to identify recombinants within highly related sequences. In brief, for each patient, a split-decomposition graph including sequences amplified from each tissue was obtained with the Neighbor Net algorithm [23]. Some graphs showed intricate networks consistent with the existence of conflicting phylogenetic signals and extensive intra-host recombination. We tested the hypothesis of intra-tissue recombination in each graph by using the pair-wise homoplasy index (PHI-test), which simulations have shown to provide a robust and reliable statistic to detect recombination [24]. The PHI statistic quantifies the incompatibility between different possible phylogenetic histories underlying the data. For each alignment of viral sequences from a tissue, we calculated the observed PHI and a null PHI distribution obtained from 1000 random alignments simulated under the null hypothesis of no recombination. An observed PHI value<5% of the values in the null distribution is evidence of a statistically significant signal for recombination. In many tissues the PHI test for recombination was highly significant (*p*<0.0001). Interestingly, while HIV-1 recombinants were detected in all patients, regardless of pathology and cause of death, they occurred in highly different proportions, ranging from 0% to 53% in different tissues (Tables 1–7). The removal of recombinant sequences significantly changed the calculated distance in only 3 out of the 29 tissue samples containing recombinants. Although recombinants were often found in tissue sequence populations with high divergence, they were also found in sequence populations with a comparatively low sequence divergence (this is especially apparent in the two lymphoma cases). Linear regression analysis comparing the number of recombinants to the diversity in each tissue showed a significant correlation of the two variables only in subject AZ (r = 0.96, p = 0.01). Therefore, we could exclude, as previously shown [22], [24], that the PHI test is biased by the extent of heterogeneity within a given data set or that higher diversity and recombination values are due to rapid viral replication in disease tissues. A high frequency of intra-host recombinant sequences was usually found in abnormal tissues associated with disease processes, including meninges, spleen and lymph nodes. No relationship between a patient's HAART history and the number of recombinants found in damaged or normal tissues was observed.

**Table 1:** Patient CX - Dementia.

Tissue	# Sequences	PHI-test p-value	# Recombinants Identified (% recombinant)	% Diversity (SE)	% Diversity without Recombinants (SE)
A-Perivascular LN	23	0.256	0	1.4 (0.1)	nc
N-Colon	24	0.900	0	2.0 (0.3)	nc
U-Periventricular White	24	0.393	0	0.8 (0.1)	nc
A-Choroid Plexus	23	0.076	0	1.6 (0.1)	nc
A-Meninges	23	**0.0(<10^−99^)**	5 (21.7)	3.9 (0.3)	3.1 (0.4)
A-Temporal Lobe	22	0.170	0	1.0 (0.1)	nc
A-Basal Ganglia	23	**1.198(10^−13^)**	5 (21.7)	4.0 (0.4)	3.7 (0.3)
A-Occipital Junction	23	**5.871(10^−11^)**	8 (34.8)	4.3 (0.4)	4.2 (0.4)
A-Occipital White Matter	24	**2.014 (10^−13^)**	9 (37.5)	2.1 (0.2)	1.8 (0.2)

**Recombination in and between tissues.** The subjects in the study are listed in Tables 1–7. Each table lists the tissues sampled, the number of sequences obtained from each tissue, the p-value for the PHI-test computed with Splitstree and the number and percent of recombinants found in the data set. The percent diversity in each tissue sequence dataset is shown with a standard error. For comparison, the percent diversity was also calculated without recombinants sequences included. Numbers highlighted in grey indicate significant p-values for recombination in the data set or a greater than 1% change in the % diversity of the sample set when the recombinant sequences were removed. Letter designations before the tissue names refer to pathology reports, A = abnormal, N = normal and U = unknown (no support for tissue pathology is written in the autopsy report). nc = No change in diversity calculation.

**Recombination tests.** The number of recombinant sequences for all tissues in each patient is shown for the PHI-test and the RDP recombinant detection methods in Table 8.

**Table 2 pone-0005065-t002:** Patient GA - Dementia.

Tissue	# Sequences	PHI-test p-value	# Recombinants Identified (% recombinant)	% Diversity (SE)	% Diversity without Recombinants (SE)
A-Meninges	23	**7.438(10^−15^)**	7 (30.4)	2.7 (0.3)	2.7 (0.3)
A-Frontal Lobe White	31	**3.492(10^−14^)**	13 (41.9)	6.2 (0.5)	**2.2 (0.2)**
A-Temporal Cortex	21	**0.002**	2 (9.5)	5.8 (0.5)	**1.5 (0.2)**
N-Lymph Node	28	**0.0(<10^−99^)**	9 (32.1)	4.7 (0.4)	4.7 (0.3)
A-Spleen	29	**0.0(<10^−99^)**	4 (13.8)	3.4 (0.3)	3.4 (0.3)

**Table 3 pone-0005065-t003:** Patient DY – Systemic Infection (including encephalitis).

Tissue	# Sequences	PHI-test p-value	# Recombinants Identified (% recombinant)	% Diversity (SE)	% Diversity without Recombinants (SE)
N-Meninges	18	**0.0(<10^−99^)**	4 (22.2)	2.8 (0.3)	2.2 (0.3)
N-Basal Ganglia	20	**0.003**	1 (5.0)	2.3 (0.3)	**0.7 (0.1)**
N-Temporal Cortex	23	1.0	0	0.3 (0.1)	nc
N-Frontal Lobe Grey	22	**2.101(10^−4^)**	1 (4.5)	2.7 (0.3)	2.7 (0.3)
N-Frontal Lobe White	24	0.188	0	0.8 (0.1)	nc
U-Liver	17	0.120	0	0.9 (0.2)	nc
A-Lymph Node	21	**1.737(10^−7^)**	5 (23.8)	3.3 (0.3)	3.0 (0.3)
A-Spleen	21	**2.320(10^−4^)**	2 (9.5)	3.0 (0.3)	2.6 (0.3)

**Table 4 pone-0005065-t004:** Patient AZ – CVD and Systemic Infection.

Tissue	# Sequences	PHI-test p-value	# Recombinants Identified (% recombinant)	% Diversity (SE)	% Diversity without Recombinants (SE)
N-Meninges	13	0.126	0	0.9 (0.2)	nc
N-Frontal Cortex	22	1.0	0	0.4 (0.1)	nc
A-Liver	17	1.0	0	0.6 (0.1)	nc
N-Lymph Node	16	**2.172(10^−4^)**	1 (6.3)	4.0 (0.3)	3.6 (0.3)
A-Spleen	19	**9.135(10^−9^)**	2 (10.5)	4.7 (0.4)	4.8 (0.4)

**Table 5 pone-0005065-t005:** Patient AM - Lymphoma.

Tissues	# Sequences	PHI-test p-value	# Recombinants Identified (% recombinant)	% Diversity (SE)	% Diversity without Recombinants (SE)
A-Left Axillary LN	22	0.336	0	0.9 (0.1)	nc
N-Diaphragm	20	0.992	0	5.8 (0.4)	nc
U-Prostate	16	1.0	0	2.3 (0.3)	nc
A-Kidney	21	1.0	0	1.2 (0.2)	nc
A-Liver 1 Biopsy	20	**0.022**	1 (5.0)	1.6 (0.2)	1.5 (0.2)
A-Liver 2 Biopsy	37	**6.378(10^−8^)**	3 (8.1)	1.1 (0.2)	1.0 (0.2)
A-Liver 3 Biopsy	20	**0.001**	2 (10.0)	1.9 (0.2)	1.5 (0.2)
A-Right Axillary LN	18	**3.344(10^−4^)**	2 (11.0)	1.6 (0.2)	1.5 (0.2)
A-Spleen	24	**5.072(10^−8^)**	7 (29.2)	2.4 (0.3)	2.2 (0.3)
A-Stomach Lymphoma	27	**7.409(10^−10^)**	4 (14.8)	1.3 (0.1)	0.3 (0.1)

**Table 6 pone-0005065-t006:** Patient IV - Lymphoma.

Tissues	# Sequences	PHI-test p-value	# Recombinants Identified (% recombinant)	% Diversity (SE)	% Diversity without Recombinants (SE)
A-Diaphragm	16	**0.003**	1 (6.3)	1.7 (0.2)	1.7 (0.2)
A-GI Tract	18	1.0	0	2.9 (0.3)	nc
A-Kidney	19	0.353	0	0.6 (0.1)	nc
A-Left Axillary LN	13	**2.912(10^−5^)**	4 (30.8)	2.4 (0.3)	2.4 (0.3)
A-Lung LN	22	**1.637(10^−13^)**	11 (50.0)	2.7 (0.3)	2.7 (0.3)
A-Periaortic LN	20	**4.722(10^−9^)**	3 (15.0)	2.5 (0.3)	2.5 (0.3)
A-Right Axillary LN	16	**2.432(10^−6^)**	4 (25.0)	2.4 (0.3)	2.3 (0.3)
A-Spleen	19	**3.624(10^−5^)**	2 (10.5)	1.9 (0.2)	1.9 (0.2)
A-Stomach	22	1.0	0	0.3 (0.1)	nc
A-Omental LN	16	0.762	0	1.8 (0.2)	nc

**Table 7:** Patient BW – Lymphoma (including leptomeningeal lymphoma).

Tissue	# Sequences	PHI-test p-value	# Recombinants Identified (% recombinant)	% Diversity (SE)	% Diversity without Recombinants (SE)
A-Meninges	22	**2.508(10^−9^)**	5(22.7)	2.5 (0.3)	2.1 (0.2)
A-Basal Ganglia	23	0.175	0	0.9 (0.1)	nc
A-Temporal Cortex	17	1.000	0	0.3 (0.1)	nc
A-Frontal Lobe Gray	21	0.091	0	0.3 (0.1)	nc
A-Frontal Lobe White	20	0.276	0	0.9 (0.2)	nc
A-Spleen	18	0.074	0	1.7 (0.2)	nc

The RDP program was also used to detect the number of recombinants in each tissue and produced similar results [25]. RDP is a useful software package for the rapid and automatic identification of recombination signals. The default setting uses seven different recombination detection methods. These are, 1) the original RDP method, 2) the Bootscan/RECSCAN method [25], [26], 3) the method applied in the program GENECONV [27], [28], 4) the MaxChi method [29], [30], 5) the Chimaera method [29], 6) the SiScan method [31] and 7) the 3SEQ method [32]. The numbers of recombinants for each subject's tissues were combined and are shown in Table 8 along with the results from the Salemi et al method. In all but one subject (AM), the RDP program identified more recombinants; therefore, it appears that the Salemi et al. method, at least in the case of intra-patient tissue recombination detection, is more conservative. This is probably because 1) some of the methods in RDP are molecular model-dependant, 2) the number of sequences within each analysis can impact results, and 3) variation within each population may alter the number of recombinants identified [33]. The significant finding from both analyses is that recombination occurred frequently, in various places along the gp120, and at various frequencies in the many tissues examined.

**Table 8:** Recombination tests.

Patient	PHI-test method	RDP method
CX	27	35
GA	35	40
DY	13	21
AZ	3	4
AM	19	10
IV	25	33
BW	5	16

### Tissue histopathology and recombination

**Figure 2:**
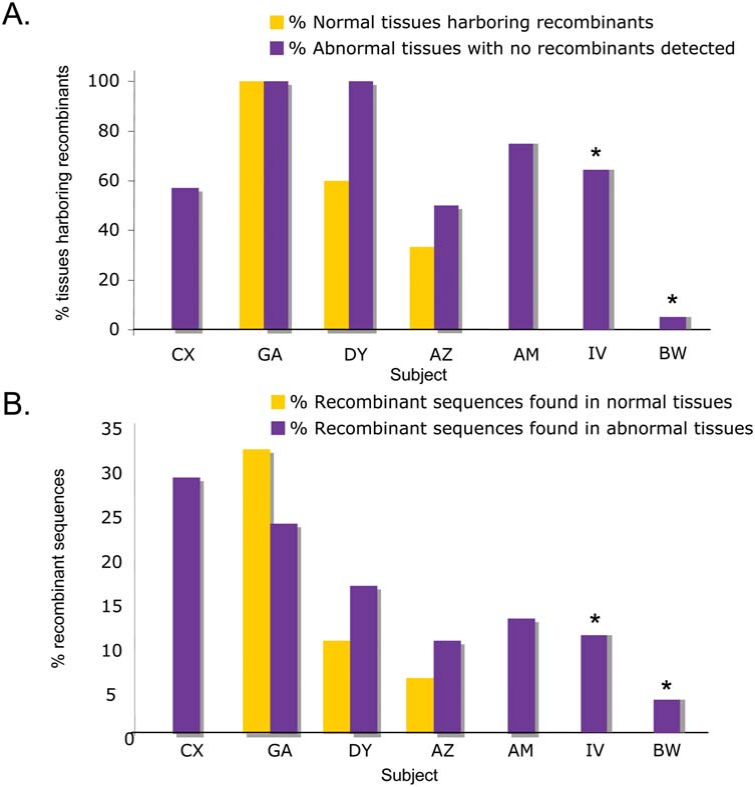
Frequency of recombination. In panel A the graph shows for each subject (x-axis), the percentage of tissues harboring recombinants (y-axis). In panel B the graph shows the percent of recombinant sequences found in normal and abnormal tissues for each subject. Tissues with normal and abnormal histopathology are indicated in yellow and purple respectively. * Only tissues with abnormal histopathology were collected from patient IV and BW.

**Figure 3:**
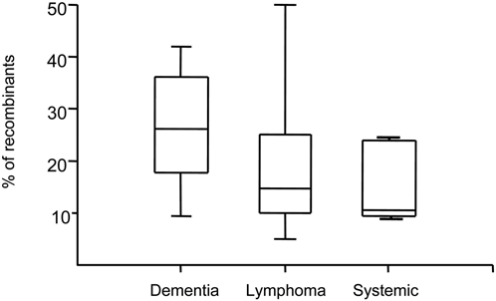
Distribution of HIV-1 recombinant sequences in subjects with different primary diseases. Each box plot shows the median, the 95-percentile distribution of the proportion of recombinants detected in tissues (y-axis) sampled from patients with different primary diseases (x-axis) and associated standard errors.

Both normal and abnormal tissues were sampled from five (CX, GA, DY, AZ, AM) out of seven patients. For two patients, IV and BW, all tissues sampled were identified as abnormal. Figure 2A shows for each subject the percentage of normal and abnormal tissues harboring recombinants. In general, the proportion of tissues harboring recombinant sequences was significantly higher among tissues with abnormal histopathology than in normal tissues (chi-square test for categorical data *p*<0.001), with the exception of one subject (GA). Statistical analysis also showed that a significantly higher proportion of recombinant sequences was detected within these abnormal tissues (chi-square test for categorical data *p*<0.001) as compared to normal tissues, with the exception, again, of subject GA (Figure 2B). Although a higher frequency of recombinant sequences was more likely to be found in tissues displaying abnormal histopathology, no significant difference was found in the extent of recombination by comparing patients with different AIDS-associated illnesses. The box-plot in Figure 3 shows that the range of recombinant sequences detected within patients grouped according to their diagnosis was largely overlapping.

Patient GA contained a relatively equal number of recombinants in one normal tissue: an axillary lymph node. This tissue also had the second highest diversity rate of all 53 tissues examined; therefore, this may be a single case where the number of recombinants was due to high viral turnover in this particular tissue at the time of death or due to other unknown factors. Further evaluation of other normal tissues from patient GA may provide evidence to establish whether the association between recombination in this case was tissue-specific or due to an overall different pattern of sequence evolution within the patient.

### Identification of recombination breakpoints

**Figure 4:**
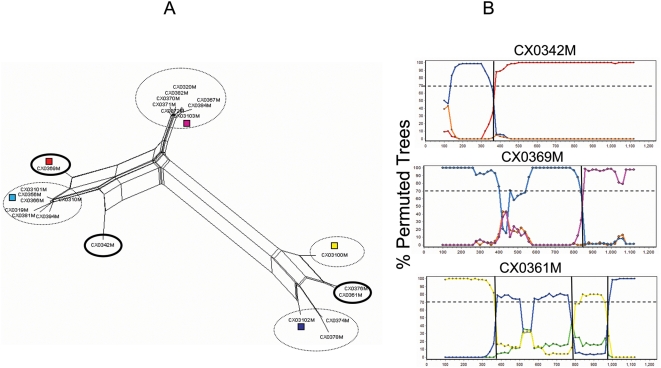
Recombination analysis of HIV-1 gp120 sequences from the meninges of subject CX. A. Neighbor-Net (NNet) obtained with the split-decomposition method and uncorrected *p*-distances for HIV-1 gp120 sequences amplified from meninges. Solid and broken circles highlight putative recombinant and parental sequences, respectively. A colored box indicates a monophyletic group of putative parental sequences to be tested in the bootscanning analysis B. Bootscanning plots of three recombinant sequences. Each bootscanning was carried out on an alignment that included a query sequence (the putative recombinant), and a set of putative parental sequences (indicated by different colors corresponding to the colored boxes in panel A. The query sequence (within the solid circles in panel A) is given at the top of each bootscanning plot. The x-axis represents the nucleotide position along the alignment; the y-axis represents the % bootstrap support for the clustering of the query sequences with one of the monophyletic groups in panel A. The crossing point between two lines of different color, indicated by a vertical solid line, specifies the assumed position of a recombination breakpoint.

While the detection of recombinant sequences is readily achievable, the detection of the precise location of a breakpoint is nearly impossible, especially in the case of highly related sequences. Still, recombination breakpoints were assessed in order to identify if potential hotspots or non-specific recombination events had occurred. The analysis was conducted using the split-decomposition networks in conjunction with visual examination of bootscans produced with Simplot [34]. As suggested by Zhang et al. [35], we used a small moving step (20 nt) for breakpoint detection; however, by varying the sliding window from 1 to 20 nt, we found that putative breakpoints could map within a genomic region of approximately 20 to 100 nucleic acids; therefore, it is important to emphasize that the breakpoints are not precise but merely provide a graphical overview of our findings. An example of our method (patient CX meninges) is given in Figure 4. Figure 4A shows the Neighbor-Net inferred tree using viral sequences from the meninges of subject CX. Putative recombinant sequences, usually located at the vertices of large splits in such networks, are highlighted within a solid circle, while broken circles indicate monophyletic groups of putative parental sequences. The bootscanning analysis shown in Figure 4B shows how putative breakpoints appear in the plot as a switch in a high bootscan value from one sequence to another related sequence. This analysis was performed for all 127 identified recombinant sequences (data available as supplemental material).

**Figure 5:**
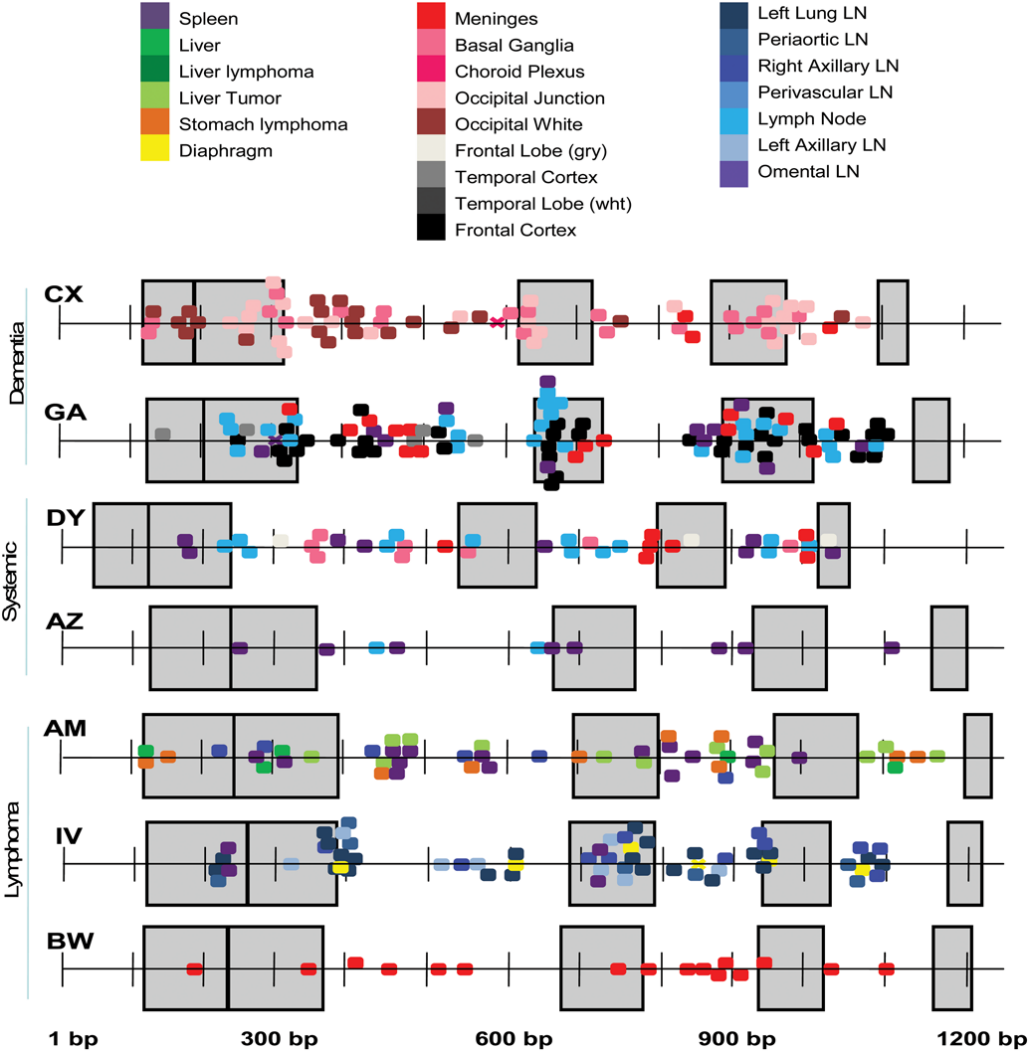
Summary of breakpoints in intra-tissue sequence populations. As noted along the bottom, the x-axis represents the 1200+ nucleotides incorporating the gp120 domain in HIV-1. The large transparent blue boxes represent the variable domains V1, V2, V3, V4 and V5 respectively. The variation in placement of the variable domains is due to natural genetic length variation between the patient's data sets and to deviation between sequencing reactions. Each plot displays the estimated location of break points found in every sequence for each patient (noted on the upper left of the plot). A colored box indicates the estimated location of a breakpoint identified in an *individual* sequence using bootscanning analysis. Each putative breakpoint is color-coded for a different tissue as shown in the figure.

Figure 5 displays a map of the putative breakpoints for all recombinant sequences in each patient. The breakpoints are color-coded by tissue. Many of the sequences contained multiple breakpoints so that the number of breakpoints on each graph is typically larger than the number of recombinant sequences listed in Tables 1–7. In most patients putative breakpoints along the gp120 appeared somewhat randomly distributed in both conserved and variable gp120 domains. However, in some cases similar breakpoints clustered to specific regions along the genome, suggesting the possibility of recombination hotspots or selective outgrowth of particular viral variants. As an example, subject GA has a large number of recombinants mapping to a similar region in the V3 domain. Subjects CX, GA and IV all have clusters of recombinant breakpoints occurring around the end of V2 domain. Alternatively, the apparent random distribution of breakpoints could be due solely to the inability to identify the precise location of breakpoints; however, with the analysis of 127 total recombinant sequences, it is unlikely that bootscanning would be so imprecise as to provide such diverse results over a region of 1200 nucleic acids.

Both dementia patients contained numerous intra-tissue recombinants with many sequences containing multiple breakpoints along gp120. These patients had putative breakpoints mapping within the first four hypervariable domains. In contrast, patients in the “systemic” disease classification contained fewer recombinant breakpoints overall. Although no brain sequences were available for the lymphoma patients AM and IV, their breakpoint results looked somewhat similar to the dementia patients. This may be due to the fact that both dementia and lymphoma are macrophage-mediated diseases. Interestingly, none of the subjects showed any recombination occurring in either the far 5′ or 3′ end of the genome, including the V5 domain.

## Discussion

Recombination plays a major role in HIV-1 evolution and represents a powerful mechanism to produce genetic diversity during the viral lifecycle. Although recombination in HIV-1 viruses has been well documented, especially between different subtypes within an individual or in the context of circulating recombinant forms in a population of infected subjects, it has been less studied in the context of its occurrence within individuals. The present study represents the most extensive investigation of HIV-1 intra-host recombination within different tissues performed to date. Seven hundred eighty gp120 sequences amplified from 53 tissues with either normal or abnormal histopathology were collected from seven patients who died of different AIDS-associated illnesses.

One characteristic of end-stage AIDS is the presence of HIV p24 expressing macrophages along with T-cell depletion. At end-stage disease, Mack et. al. [36], found that in a large variety of autopsy tissues classified as diseased tissues, including lymph nodes, spleen and brain, with amplifiable amounts of DNA, p24 antigen staining was predominantly localized to macrophages interspersed in a background of p24-negative lymphocytes. This type of staining was not seen in non-diseased tissues in HIV positive patients [36]. We found a similar staining pattern in the tissues used for this study. The higher frequency of recombinant sequences consistently found in tissues with abnormal histopathology is likely explained by the fact that such tissues display increased macrophage proliferation due to an inflammatory response. HAART therapy, which was given to four of the seven patients prior to death, appeared to have little effect on overall recombination rates. This is not unexpected, since macrophages are one of the most important cellular reservoirs sustaining virus replication during HAART [37]. In fact, several studies have implicated HAART therapy in the development of metabolic lipid dysfunction and other disorders that can lead to tissue abnormalities [38]–[40]. The results strongly implicate macrophages as the primary producers of recombinants in damaged tissues. Tissue samples taken over time from an animal model would confirm if a similar association between recombination and abnormal tissues are typical in early and mid-stage, rather than only at end-stage disease.

HIV integration within host cell genomic DNA is a required step of the viral infection cycle. HIV integration site mapping and laser capture micro dissection analysis of infected macrophages have shown that viral integration usually occurs within introns of genes related to or near cellular activation loci [36]. It is interesting to note that the brain specimens used to map insertion sites in Mack et al. [36] were also analyzed in the current study (patient CX), demonstrating that high recombination rates occur within brain macrophages that contain inserted forms of HIV. Sequences from patient CX were also analyzed using a phylodynamic approach [41]. This study showed that activated macrophages migrate between HIV infected brain tissues and especially to areas of the brain where there is an abundance of tissue damage. Persistent macrophage activation is associated with an inhibition of apoptotic signals, giving end-stage HIV-infected macrophages a survival advantage, the ability to act as a continuous source of HIV and to serve as a long-term reservoirs/sites of viral recombination [42]. Furthermore, tissue macrophages co-infected with opportunistic pathogens such as *Mycobacterium Avian Complex* (MAC) or *Pneumocystis carinii* dramatically increase viral production and the likelihood of macrophage-mediated tissue destruction [43].

The occurrence of HIV-1 recombination *in vivo* can be explained by the ability of the nascent viral strand to switch RNA templates during reverse transcription [44], and it necessitates super-infection of the target cell with two or more viruses, each carrying a different HIV-1 genome. Certain cell-types may be more prone to multiple infections than others. For example, cells that live longer or tissue sites of continual feeding of new viral populations would be more likely to be super-infected. The continuous recruitment of macrophages at infection sites and their long lifespan makes them the perfect target for super-infection. While the turnover of HIV-infected activated T-cells is 2–3 days, infected macrophages survive considerably longer, as discussed above, which increases both the probability of super-infection and the probability of recombination once super-infection has occurred. Our finding of a large number of recombination breakpoints distributed across the gp120 envelope protein in abnormal tissues with high levels of replication-competent macrophages is consistent with *in vitro* studies, which showed that while a single round of viral replication in T-lymphocytes in culture generated an average of nine recombination events, the infection of macrophages led to approximately 30 crossover events per cycle [11].

Other studies have identified macrophages as a source of the production of recombinant viruses [11], [45], [46], but their role as a major contributor to this process remains a subject of debate. The combined results from our study demonstrate an increase in the potential for macrophage-mediated immune evasion during HIV disease because: 1) abnormal or damaged tissues commonly occur during prolonged HIV disease, 2) damaged tissues recruit macrophages that are clearly a replication site for HIV, whereas normal tissues do not, 3) as activated macrophages accumulate within abnormal tissues, they may become super-infected, thus increasing in the potential for the generation of recombinants, 4) any HIV-associated disease process or HAART-associated side effect that generates tissue damage has the potential to increase the production of recombinants, 5) the degree of recombinants produced within an individual may increase during HIV- or HAART-associated tissue damage within an individual. Importantly, if macrophages are a continued reservoir for the generation of HIV-1 intra-patient recombinant sequences *in vivo*, then they are also source of continued viral evolution and diversification over time. The current study provides additional evidence that successful eradication of HIV-1 is unlikely to be achieved unless new therapeutic approaches specifically targeting tissue macrophages are developed.

## Materials and Methods

### Biomaterial

Frozen autopsy tissues from patients and accompanying pathology records were obtained from the University of California at San Francisco AIDS and Cancer Resource (ACSR) (url: http://acsr.ucsf.edu). The ACSR is a National Cancer Institute Funded tissue banking program that obtains tissues from patients after appropriate consent and the application of a de-identification procedure before sending the tissues out to ACSR approved investigators. Clinical histories are similarly handled in a de-identified manner. The patient designations used throughout this study do not relate to the patients, but were randomly generated as shorthand used by technicians who perform the studies. The ACSR is recognized by the Office of Biorepositories and Biospecimen Research at the National Institutes of Health as being HIPAA compliant. Additionally, all material was obtained under approval from the UCSF committee on human research. Although every attempt was made to utilize similar tissues across the subjects in the study, this was often difficult. Two subjects, patients AM and IV, who died due to AIDS-related lymphoma, contained no amplifiable DNA within several brain tissues examined.

### Characterization of patient specimens

All frozen tissues had parallel fixed tissues available for hematoxylin and Eosin staining as well as immunohistochemical staining. Tissues were stained with the tissue macrophage specific antibody, anti-CD68, with recent tissue migrant macrophage specific antibody, anti-MAC387 and with anti-HIVp24. All antibodies were obtained from DAKO and were used as suggested in the accompanying product insert and as previously described [36].

### HIV PCR, cloning and sequencing

Genomic DNA was extracted from 10–30 mg of each tissue using the QIAmp DNA Mini Kit from Qiagen according to the manufacturer's instructions. A 3.3 kb HIV fragment, extending from env to the 3′LTR was amplified by PCR using the primers EnvF1 (AAC ATG TGG AAA AAT AAC ATG GT) and NefR1 (ACT TDA AGC ACT CAA GGC AA) under the following conditions: an initial denaturation step of 94°C for 5 min followed by 35 cycles of 94°C for 30 sec, 55°C for 30 sec, 68°C for 3 min 20 sec, and a final extension at 68°C for 8 min, in 50 µl volume using 600 ng of template DNA, 10 µl of 10× buffer (Invitrogen), 10 mM deoxynucleoside triphosphates (Invitrogen) 60 µM of each primer, and 2.5 units of Invitrogen Platinum-Taq polymerase. Products were cloned into the pCR2.1-TOPO vector according to the manufacturer's instructions and the resultant colonies were screened for the proper insert using an identical PCR protocol. Sequencing was performed on approximately 20–40 clones derived from each tissue by ELIM Biopharmaceuticals, Hayward, CA.

### Data screening and management

Because of the large amount of sequence data produced for the study and the risk of sequence contamination or PCR over-representation at the many levels of experimentation, a computational pipeline for screening the entire data base of sequences was developed. The algorithm involved a feedback method from the examination of 3.3 kb alignments and phylogenetic analysis. The method progressed with an initial set of approximately 20 sequences from a single tissue. Any 3.3 kb sequences with 100% identity were removed from the sample set in order to avoid over-representation of a single variant by the polymerase chain reaction. Sequences that contained unusual base substitution rates or large amounts of ambiguous sites were also discarded. Next, in order to identify potential inter-tissue contamination, a maximum-likelihood phylogenetic tree was generated from different tissues from the same patient. When sequence tissue populations were non-compartmentalized, 15 additional HIV DNA clones were generated for each non-compartmentalized tissue from the original DNA sample. A second generation of independent clones was sequenced and the distribution of sequences among the first generation clones was compared to that of the second generation. If the case arose where the second round varied significantly from the first, a third set of sequences was obtained and to determine if the results were reliable. The examination of multiple PCR reactions for specific tissues enabled the confirmation of sequence integrity in the database. Screening for inter-patient contamination was also accomplished using standard phylogenetic methods. The protocol was designed to reduce the possibility that the data set contained unreliable sequences due to over-amplification of specific viral variants, problematic sequences due to PCR contamination, sample mislabeling, inter-subject contamination, intra-subject contamination or sequences that clustered with significant variance over independent samplings in a phylogenetic tree. An automated version of the phylogenetic clustering program is available at www.bioinfoexperts.com/icarus. The cautious selection of very high quality data is necessary in a study such as this where final analysis is contingent upon sequence integrity. PCR limiting dilution assays, as suggested by Rodrigo et al. [47] are not feasible for the development of a sequence data base of this size.

The gp120 domain was identified and retrieved from the 3.3 kb fragment using HIVbase software (http://www.hivbase.com) [48]. The CLUSTAL algorithm [49] was used to generate multiple sequence alignments. For final gp120 alignments, a slightly modified protocol developed by Lamers et al. [50] using glycosylation motifs as anchors in the alignment process was used to maximize positional homology in gap-rich regions [50]. Sequence data were deposited in Genbank.

### Analysis for intra-tissue recombination

Several algorithms were combined to analyze data sets and individual sequences for recombination [22]. Our first goal was to identify putative recombinants within each tissue. Aligned sequences were imported into Splitstree, (www.splitstree.org) [24] and a preliminary network using the Neighbor Net algorithm [23] was obtained. Splitstree currently contains one of the more robust methods for determining the likelihood of recombination in a set of aligned sequences [24], called a PHI-test. A PHI score with a *p*-value<0.05 shows with significance that recombination occurs in the data set. When a set of sequences produced a complex network, along with a *p*-value less than 0.05, putative recombinants were identified by filtering them from the data set and recalculating the PHI-test to check whether the removal of such sequences significantly increased the *p*-value. The removal of all recombinants from the data set eventually increased the PHI-test *p*-value to a level of no significance. Once the *p*-value for the Phi-test was >0.05, each sequence that was removed was reinserted individually into the Neighbor Net to make sure that it significantly impacted the results of the PHI-test.

The program RDP was also used to identify the number of recombinants in each tissue using the same sequence alignments as in the previous analysis [51]. All recombinants identified for each subject were combined and are shown in Table 8. Default settings in the program were used for the analysis.

To identify individual putative breakpoints in each recombinant sequence we used a sliding-window, bootscanning approach, which computes a bootstrapped maximum-likelihood phylogenetic tree for overlapping segments of the alignment (in our case, each 20 nucleotides) [52]. As each segment is calculated, the percent bootstrap value with its closest relative sequence remains high until a breakpoint is found in the compared data set. The putative breakpoints appear in the plot as a switch in a high bootscan value from one sequence to another related sequence. The intersection of the bootscan plots estimates the location of the breakpoint. Simplot software (ver3.5.1)[34] was used for the bootscanning analysis (Figure 1). Simplot bootscans for all recombinants are available as supplemental material. Putative breakpoints for all recombinants were mapped as in Figure 5. These breakpoints were not always precise; in-depth analysis showed that bootscanning could sometimes map breakpoints into regions<10 nt long, whereas other times the putative breakpoint could have occurred anywhere in a region over 200 nucleotides in length.

### Statistical analysis

We used a Chi-squared test for categorical data to test whether viral recombination across patients tended to occur with a significantly greater frequency in normal or abnormal tissues (test 1), and whether the frequency of recombinant sequences was significantly higher in tissues with abnormal histopathology than in normal tissues (test 2). The Chi-squared test was performed in SigmaStat 3.0 with and without the Yate's Correction Factor.

## Supporting Information

Supporting Materials S1 (0.03 MB DOC)
