# Supplementary material for: Extensive HIV-1 Intra-Host Recombination Is Common in Tissues with Abnormal Histopathology
Source: PLoS One. 2009 Mar 31;4(3):e5065. doi: 10.1371/journal.pone.0005065 (PMC2659430; doi:10.1371/journal.pone.0005065)
Supplement: Supporting Materials S1 — (0.03 MB DOC) [file pone.0005065.s001.doc]

**SUPPORTING MATERIAL**

**CASE STUDIES**

Patient CX was a male with a two-year history of AIDS dementia prior to death and before the availability of HAART. One month before death he became more severely demented and cachectic, with seizures and arrest noted as the cause of death. Autopsy reports revealed that all lymph nodes were lymphocyte depleted with multi-nucleated giant cells (MNGCs) present. His lungs were occupied with necrotizing pneumonia. Throughout the brain grey and white matter there was marked astrocytosis, microgliosis and macrophage infiltration. The frontal cortex contained MNGC's and the meninges were fibrotic. The GI tract was normal. His only known history of any opportunistic infections other than dementia was CMV retinitis of the left eye.

Patient GA was a male with severe dementia who died of pneumonia after treatment with HAART. The patient had a history of thrombosis and idiopathic thrombocytopenic purpura. At death his CD4 count was 400. The autopsy revealed an enlarged spleen, liver with micro abscesses, and severe atherosclerosis. The lymph nodes were normal. There was perivascular inflammation throughout his brain consistent with HIVE, however HIV p24 stains were negative and lymphocytes were present along with macrophages in the perivascular infiltrates. The patient had no evidence of lymphoma; however he showed indications of an intense systemic infection that may have involved his brain. The patient stopped HAART treatment during the month prior to his final stay in the hospital.

Patient DY was a male that, despite HAART therapy, died with a viral load of greater than 7 million copies of HIV/ml of plasma. He was profoundly demented and had *Mycobacterium avium* infection (MAC) for a year prior to death. At autopsy his brain did not show the typical histology of patients with classical HAD; the tissue was essentially normal with no inflammation of the meninges, cortex or basal ganglia. MNGCs and slight macrophage infiltration into the white matter was consistent with a low level HIV encephalitis. The patient’s lymph nodes and spleen were enlarged and infiltrated with MAC. The liver was normal.

Patient AZ was a female on HAART with chronic hepatitis C and cardiovascular disease leading to a gastrointestinal bleed and death. She stopped taking HAART one week prior to death. Her spleen was depleted of lymphocytes, whereas her lymph nodes were not. The brain was normal except for widespread atherosclerosis. The autopsy revealed metastatic adenocarcinoma of unknown origin primary in the liver.

Patient AM was a male that died without HAART therapy. Two months prior to death he presented with lymphodenopathy, peripheral edema with a supraclavicular lymphnode biopsy showing follicular hyperplasia. He had a two week history of weight loss and fever with a left painful axillary lymph node and palpable nodes elsewhere. On autopsy there were large-cell lymphoma tumor nodules on the stomach, within the liver, encasing the pancreas, within the spleen, in all lymph nodes and the right kidney. There was no evidence for tumors in the prostate or diaphragm. The patient had no sign of brain abnormalities.

Patient IV was a male that died without HAART therapy. Two weeks prior to death he presented with ascites. One week later he developed diffuse adenopathy and fevers. High-grade lymphoma was diagnosed in the bone marrow 1 day prior to death which occurred due to respiratory distress. On autopsy, high grade EBV+ lymphoma was identified in all nodes, spleen, throughout the peritoneal cavity encasing the stomach, intestines, kidney, and diffusely infiltrating the diaphragm and omentum. The brain showed multinucleated giant cells and perivascular macrophage cuffing consistent with HIV associated encephalopathy.

Patient BW was a male who died with disseminated lymphoma and Cytomegalovirus infection. Lymphoma was present in his lymph nodes and spleen along with significant leptomeningeal lymphoma. The basal ganglia, frontal and temporal lobes were populated with multinucleated giant cells, consistent with severe HIV encephalitis. The cause of death was renal failure secondary to terminal systemic lymphoma, CMV infection and HIV encephalitis. He had recently been treated for lymphoma and was on HAART, but all medications were stopped several days before death.

Using a newly developed artificial neural network approach, as described in SL Lamers , et al. (2008, Predicting HIV R5, X4 and dual tropic phenotype using evolved neural networks. IEEE/ACM Transactions In Computational Biology and Bioinformatics 5(2):291-300), predicted co-receptor usage of all viral sequence from subjects CX, GA, AM, IV, BW, and AZ resulted CCR5, while DY’s sequences were determined to be dual tropic (i.e. utilizing both the CCR5 and CXCR4 co-receptor).
